# Application of laryngeal mask airway anesthesia with preserved spontaneous breathing in children undergoing video-assisted thoracic surgery

**DOI:** 10.3389/fped.2023.933158

**Published:** 2023-03-10

**Authors:** Jinjin Huang, Wenfang Huang, Jie Zhang, Zheng Tan, Dongpi Wang

**Affiliations:** ^1^ Department of Anesthesiology, Children's Hospital, Zhejiang University School of Medicine, National Clinical Research Center for Child Health, Hangzhou, China.; ^2^ Department of Thoracic Surgery, Children's Hospital, Zhejiang University School of Medicine, National Clinical Research Center for Child Health, Hangzhou, China

**Keywords:** anesthesia, video-assisted thoracic surgery, enhanced recovery after surgery, pediatrics, paravertebral block

## Abstract

**Purpose:**

To investigate the feasibility and safety of non-intubated general anesthesia with spontaneous breathing combined with paravertebral nerve blocks (PVNB) in young children undergoing video-assisted thoracic surgery (VATS) and to determine its significance for rapid recovery after pediatric thoracic surgery.

**Methods:**

The data of 46 children aged 6–36 months with an American Society of Anesthesiologists status of I–II who underwent elective VATS under general anesthesia were retrospectively analyzed. Of these patients, 25 underwent non-intubated general anesthesia with spontaneous breathing combined with PVNB (non-intubation group), and 21 received conventional intubated general anesthesia combined with local infiltration anesthesia (intubation group). The following perioperative parameters were compared between the two groups: heart rate (HR), mean arterial pressure, saturation of pulse oximetry (SpO_2_), partial pressure end-tidal carbon dioxide, time from the completion of the operation to extubation or removing laryngeal masks, time to first feeding after the operation, length of postoperative in-hospital stay, incidence of postoperative complications, and hospitalization expenses.

**Results:**

The operations were completed successfully in both groups. When the non-intubation group was compared with the intubation group, the minimal SpO_2_ level during the surgery was higher (93% vs. 88%, *P* < 0.001), which might indicate better oxygenation. There was no significant difference of the duration of surgery and intraoperative blood loss between two groups. Compared to the intubation group, the duration of anesthesia (*P* = 0.027), time from the completion of the operation to extubation (*P* < 0.001), time to the first feeding after surgery (*P* < 0.001), and length of postoperative in-hospital stay (*P* < 0.001) were significantly reduced in the non-intubation group. The incidence of postoperative complications was not significantly different.

**Conclusions:**

Non-intubated general anesthesia with spontaneous breathing combined with PVNB is safe and feasible in young children undergoing VATS and can promote rapid recovery in young children undergoing thoracoscopic surgery.

## Introduction

At present, video-assisted thoracic surgery (VATS) is relatively well-developed in China and has been widely used for the treatment of lung bullae, pulmonary cysts, and isolated mediastinal tumors and for sleeve resection, wedge resection, segmentectomy, lobectomy, and thymectomy (1–5). The collapse of the ipsilateral lung, which allows surgeons to operate in a limited space, is essential for VATS. A double-lumen endotracheal tube can be used in adults for one-lung ventilation (OLV). However, double-lumen endotracheal tubes suitable for children, especially for infants, are not available in the Chinese market. Endobronchial blocker (BB) placement is the preferred and most frequently used lung separation technique in children (6–9). Due to the immaturity of organ development and the small body size of infants, the risk of lung separation in these children is higher than that in preschool-age or older children. In recent years, to avoid the injuries and risks related to intubated anesthesia, some medical institutions have reported their experience with non-intubated general anesthesia with spontaneous breathing in thoracic surgery for adults. These thoracic surgeries have all been performed with regional nerve blocks [epidural nerve block, paravertebral nerve blocks (PVNB), or intercostal nerve block] when patients are awake or under sedation (2, 10, 11). For preschool children, even simple operations cannot be performed under local anesthesia only if with deep sedation or general anesthesia. Wei et al. had combined non-intubated anesthesia and PVNB to enhance recovery in children aged 3–8 years undergoing VATS (12).

We tried an anesthetic technique for VATS to achieve a safer and faster postoperative recovery with fewer postoperative complications in infants and young children. The technique of non-intubated general anesthesia with spontaneous breathing combined with PVNB was performed in 25 infants from May 2019 to June 2020 in our department. In this study, we introduce the experience from the retrospective data of these patients.

### Patients and methods

**Figure 1:**
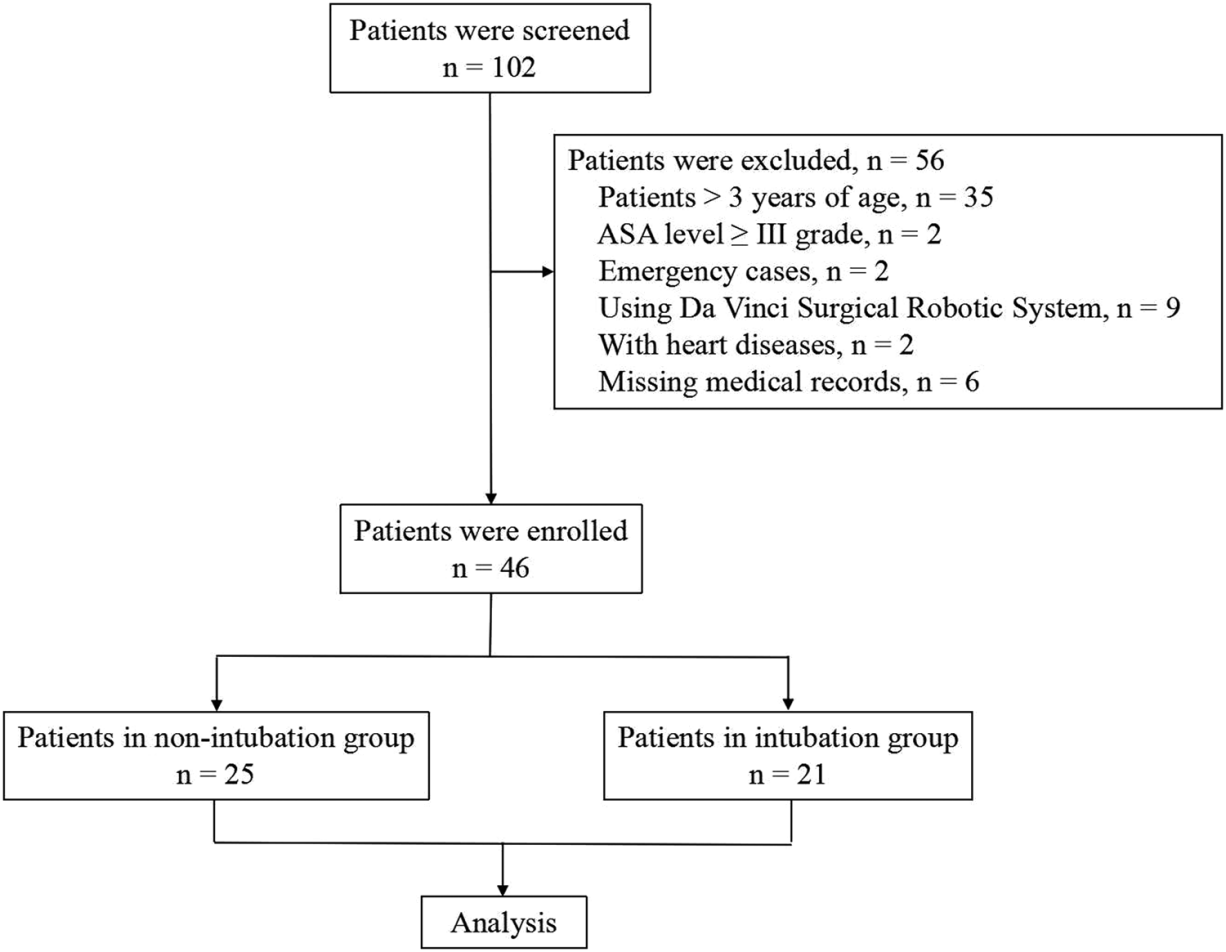
Patient inclusion and exclusion criteria for retrospective analysis.

This was a single-center retrospective study that was approved by the Ethics Committee of Children's Hospital, Zhejiang University School of Medicine (approval number: 2020-IRB-199). The clinical data of young children undergoing elective VATS from May 2019 to June 2020 were collected from the medical records. Patients ≤3 years of age; performing segmentectomy, lobectomy, or wedge resection; and with American Society of Anesthesiologists (ASA) level between I and II grade were included. Patients >3 years of age, ASA level ≥ III grade, emergency cases, using Da Vinci Surgical Robotic System, with heart diseases, and with missing medical records were excluded from the database. According to the different anesthesia methods, the enrolled children were divided into an intubation group and a non-intubation group for retrospective analysis (Figure 1).

### Anesthesia and surgical management

All of the children were routinely fasted before the operation and were transferred to the operating room with a peripheral intravenous catheter that was established in the ward. The children were given dexmedetomidine (2 µg/kg) nasal drops in the waiting area 30 min before the operation and received intravenous midazolam (0.1 mg/kg), propofol (1–2 mg/kg), and nalbuphine (0.2 mg/kg) for deep sedation after entering the operating room. Mean arterial pressure (MAP), electrocardiogram (ECG), saturation of pulse oximetry (SpO_2_), partial pressure end-tidal carbon dioxide (P_ET_CO_2_), and bispectral index (BIS) were monitored. Arterial blood gas (ABG) was monitored intermittently during the operation.

In the non-intubation group, the patient was placed in lateral decubitus position; the operative field was routinely disinfected and draped. Thoracic PVNB was performed at the site 1–1.5 cm lateral to the midline of the spine (corresponding intercostal space: T4–T5 or T5–T6) under ultrasound guidance. Ropivacaine 0.2% (0.5 ml/kg) was injected after the absence of cerebrospinal fluid or blood at withdrawal was confirmed. Oxygen (4 L/min) was given *via* the anesthesia mask during the operation. After the PVNB was completed, remifentanil (0.5–1.0 µg/kg) and propofol (1–2 mg/kg) were slowly injected intravenously. When the BIS score dropped to 40–60 and the lower jaw was slightly relaxed, an appropriate laryngeal mask (Ambu^@^ Aura-i^TM^ # 1.5 for children of body weight in 5–10 kg; # 2 for 10–20 kg) was placed and connected to an anesthesia machine with the mode set to continuous spontaneous ventilation. Continuous intravenous infusion of propofol (30–80 µg/kg/min), dexmedetomidine (0.2–0.5 µg/kg/h), and remifentanil (0.05–0.1 µg/kg/min) was maintained *via* an intravenous pump. No muscle relaxants were given, and the speed of anesthetics was adjusted according to the patient's condition to maintain spontaneous breathing. The BIS score was maintained between 40 and 60. When the respiratory rate was less than 12 breaths/min, P_ET_CO_2_ >70 mmHg, or SPO_2_ <90%, temporary manual assisted breathing was performed to restore spontaneous breathing and improve other indicators by adjusting the depth of anesthesia.

In the intubation group, after the children were deeply sedated and vital signs were monitored, fentanyl at 2.0–3.0 µg/kg and rocuronium at 0.6–0.9 mg/kg were injected intravenously. After the induction of anesthesia, an external blocker (Hangzhou Tappa EBT0105, BB 5 Fr) was placed, and the tracheal intubation was performed. Mechanical ventilation was performed to control breathing with intermittent positive pressure ventilation. OLV commenced with a tidal volume of 6 ml/kg with positive end-expiratory pressure (PEEP) at 6 cm H_2_O, while two-lung ventilation (TLV) commenced with 8 ml/kg with PEEP at 3 cm H_2_O. The set inspired oxygen fraction was maintained at 1.0 during one-lung ventilation. The blocker was adjusted under fiber-optic bronchoscopy to implement OLV on the nonsurgical side. During the surgery, propofol, remifentanil, and dexmedetomidine were intravenously administered at the same dosage that was used in the non-intubation group. Local infiltration anesthesia, ropivacaine 0.2% injected 1 mL per incision, was performed before skin incision. The BIS score was maintained at 40–60, and muscle relaxants were added intermittently.

For surgery, every child was placed in lateral decubitus position (on the healthy side). VATS operation was performed by three ports. One port was made between the fifth and sixth intercostal space along the anterior axillary line. One port was made at the eighth intercostal space along the posterior axillary line. Another port was made at the sixth or eighth intercostal space in subscapularis muscle. In both groups, iatrogenic pneumothorax with carbon dioxide was used to collapse the lung on the operative side. All patients underwent segmentectomy or lobectomy of the lung where the lesion was located. In the non-intubation group, after the lung was collapsed, 5 mL of 1.0% lidocaine was sprayed on the surface of the lung and the hilar area, and vital signs were observed for 30 s. The operation was started after the patient's vital signs were stable. A cutting stapler or ultrasonic knife was used to remove the lung segment or lobe where the lesion was located under video assistance in both groups. After the operation was completed, TLV was offered with lung recruitment maneuvers: sustained airway pressure of 30 cm H_2_O for 15–20 s. The cutting surface was free of air leakage upon forced lung expansion. After the completion of hemostasis and a correct tally of surgical instruments, the chest was closed layer by layer without drains.

Vital signs were collected during surgery at the following time points: after sedation (T_0_), after intubation or laryngeal mask placement (T_1_), 5 min after the start of the operation (T_2_), before the completion of the operation (T_3_), and 24 h after the operation (T_4_). The following data were collected: operation time, duration of surgery, duration of anesthesia, intraoperative blood loss, postoperative extubation (laryngeal mask) time, time to first feeding after surgery, length of postoperative in-hospital stay, hospitalization expenses, and postoperative complications. The examination time of postoperative complications was the second to third day postoperatively. We followed up the complications until discharge. The Faces, Legs, Activity, Cry and Consolability (FLACC) Behavioral Pain Assessment Scale was used for postoperative pain assessment (13, 14).

### Statistical methods

IBM SPSS 19.0 software was used for data analysis. Quantitative data with a normal distribution are expressed as mean ± standard deviation (mean ± SD). The independent-samples *t*-test was used to determine differences between the groups, and the paired-samples *t*-test was used to determine differences within a group. Non-normally distributed quantitative data are expressed as median (interquartile range) (median, IQR), and the rank sum test (Mann–Whitney *U* test) was used to determine differences between the groups. Count data are expressed as the number of cases and percentages, and the chi-square (*χ*^2^) test was used to determine differences between the groups. *P* < 0.05 was considered statistically significant.

## Results

During the study period, a total of 46 ASA I–II children underwent VATS. Of these patients, 25 underwent non-intubated general anesthesia with preserved spontaneous breathing combined with PVNB (non-intubation group). In the non-intubation group, there were 12 males and 13 females, with an average age of 1.0 ± 0.5 years and a weight of 9.3 ± 2.0 kg; there were 14 cases of pulmonary sequestration (PS) and 11 cases of bronchogenic cyst (BC). Conventional intubated general anesthesia was performed in 21 patients (intubation group). In the intubation group, there were 9 males and 12 females, with an average age of 1.1 ± 0.3 years and weight of 9.6 ± 1.2 kg; there were 3 cases of PS and 18 cases of BC. The children in both groups had no history of surgical trauma and no comorbid diseases. All children underwent chest x-ray or chest computed tomography examination before surgery to determine the location and nature of the lesion. Preoperative cardiac color ultrasound and electrocardiogram showed no obvious surgical contraindications. There was no significant difference in general data, such as sex, age, weight, duration of surgery, and intraoperative blood loss, between the two groups. The diagnosis was PS or BC, and all patients underwent lung segmentectomy or lobectomy (Table 1).

**Table 1:** Patients’ characteristics.

	Intubation group (*n* = 21)	Non-intubation group (*n* = 25)	*p*-value
Sex (male/female)	9/12	12/13	0.727
Age (years)	1.1 ± 0.3	1.0 ± 0.5	0.460
Weight (kg)	9.6 ± 1.2	9.3 ± 2.0	0.519
Diagnosis (PS/BC)	3/18	14/11	0.004
Surgery (Seg/Lob)	13/8	11/14	0.226
Segmentectomy (left/right)	7/6	6/5	0.105
Lobectomy (left/right)	5/3	11/3	0.624
Intraoperative blood loss (mL)	8 ± 5	5 ± 3	0.081

Note: Data are presented as number of patients (%) or mean ± SD.

PS, pulmonary sequestration; BC, bronchogenic cyst; Seg, segmentectomy; Lob, lobectomy.

In both groups, sufficient depth of anesthesia was achieved, the operation went smoothly, and the perioperative vital signs were stable. Compared with the intubation group, the non-intubation group had a lower HR at T_1,2,4_, a lower MAP at T_1–4_, a higher SpO_2_ at T_0–2_, a higher P_ET_CO_2_ at T_3_, and a higher BIS score at T_3_. Compared with T_1_, the end-tidal carbon dioxide (P_ET_CO_2_) at T_3_ decreased in the intubation group, while it increased at T_2–3_ in the non-intubation group. There was no significant difference in the highest P_ET_CO_2_ between the two groups, but the lowest SpO_2_ in the non-intubation group was higher than that in the intubation group (93% vs. 88%, *P* < 0.001). The two groups had similar duration of surgery. Compared with the intubation group, the non-intubation group had a shorter duration of anesthesia (Table 2).

**Table 2:** Comparison of perioperative vital signs between the two groups.

	Time points	Intubation group (*n* = 21)	Non-intubation group (*n* = 25)	*p*-value
HR (bpm)	T_0_	127 ± 14	121 ± 8	0.123
	T_1_	134 ± 20*	117 ± 9	0.002
	T_2_	125 ± 16	108 ± 8*	<0.001
	T_3_	125 ± 13	118 ± 15	0.118
	T_4_	131 ± 7	121 ± 5	<0.001
MAP (mmHg)	T_0_	64 ± 7	62 ± 8	0.025
	T_1_	64 ± 5	60 ± 6	0.025
	T_2_	75 ± 6*	62 ± 8	<0.001
	T_3_	71 ± 8*	65 ± 7	0.014
	T_4_	73 ± 10*	65 ± 4	0.002
SpO_2_ (%)	T_0_	98 ± 2	99 ± 1	0.04
	T_1_	97 ± 3*	99 ± 1	<0.001
	T_2_	97 ± 3	99 ± 1	0.011
	T_3_	99 ± 1	99 ± 1*	0.864
	T_4_	99 ± 1	99 ± 1	0.057
P_ET_CO_2_ (mmHg)	T_1_	38 ± 5	38 ± 5	0.732
	T_2_	40 ± 7	39 ± 6**	0.841
	T_3_	34 ± 4**	44 ± 5**	<0.001
BIS	T_0_	71 ± 3	71 ± 3	0.599
	T_1_	52 ± 6*	54 ± 6*	0.319
	T_2_	49 ± 7*	58 ± 7*	<0.001
	T_3_	54 ± 7*	61 ± 5*	<0.001
Peak P_ET_CO_2_ (mmHg) during surgery	52 (46, 61)	55 (49, 58)	0.963
Cases with intraoperative SpO_2 _< 90% (*n*)	15 (71.4%)	6 (24%)	0.01
Lowest intraoperative SpO_2_ (%)	88 (83, 91)	93 (90, 96)	<0.001
Duration of surgery (min)	58 ± 24	50 ± 8	0.141
Duration of anesthesia (min)	121 ± 31	102 ± 25	0.027

HR, heart rate; MAP, mean arterial pressure; SpO2, saturation of pulse oximetry; PETCO2, partial pressure end-tidal carbon dioxide; BIS, bispectral index; T_0_: after sedation; T_1_: after intubation or laryngeal mask placement; T2: 5 min after the start of the operation; T_3_: before the completion of the operation; T_4_: 24 h after the operation.

Data are presented as number of patients (%), mean ± SD, or median (interquartile range).

* *P* < 0.05 compared with T_0_.

** *P* < 0.05 compared with T_1_.

In the non-intubation group, temporary manual assisted ventilation was performed for six patients (24%) because of SpO_2_ <90%. SpO_2_ improved after transient manual assisted ventilation. One patient (4%) had coughing during the operation, which caused significant mediastinal flutter and affected the operation. Therefore, anesthesia machine-assisted ventilation was performed after anesthesia was deepened to ensure a smooth operation. In the intubation group, SpO_2 _< 90% during the operation was observed in 15 patients (71.4%), and breathing parameters returned to normal after sputum suction and adjustment of ventilation parameters. Displacement of the BB after positioning or during surgery occurred in six patients (28.6%), which caused the failure of one-lung ventilation, and the lung on the operation side was expanded. These operations were completed under TLV. There were no perioperative deaths in the two groups.

When the non-intubation group was compared with the intubation group, the time from the completion of the surgery to extubation (*P* < 0.001), the time to first feeding after surgery (*P* < 0.001), and the length of postoperative in-hospital stay (*P* < 0.001) were significantly reduced. The FLACC pain score of the two groups was below 3 on the first postoperative day, and the analgesia effect was satisfactory. In the non-intubation group, there were two cases (8%) of nausea and eight cases of pneumothorax (32%), and the complication rate was 40%. In the intubation group, there were seven cases of pneumothorax (33.3%), six cases of pleural effusion (28.6%), and one case of atelectasis (4.8%), and the complication rate was 66.7%. The patient with atelectasis also had a severe lung infection. There was no statistically significant difference in the overall incidence of complications between the two groups (*P* = 0.071) (Table 3).

**Table 3:** Postoperative recovery.

	Intubation group (*n* = 21)	Non-intubation group (*n* = 25)	*p*-value
Time from completion of surgery to extubation (min)	141 (109–202)	11 (3–15)	<0.001
Time to first feeding after surgery (min)	330 (299–379)	45 (35–90)	<0.001
Postoperative complications	14 (67%)	10 (40%)	0.071
Nausea	0	2	
Pneumothorax	7	8	
Pleural effusion	6	0	
Atelectasis	1	0	
FLACC pain scale on the first postoperative day	0 (0–1)	0 (0–1)	0.242
Length of postoperative hospital stay (days)	6.7 ± 1.3	3.7 ± 0.5	<0.001

FLACC, Faces, Legs, Activity, Cry and Consolability.

Data are presented as number of patients (%), mean ± SD or median (interquartile range).

## Discussion

In recent years, VATS has been performed widely in children with diseases of the esophagus, lung, mediastinum, and so on. Lung isolated technique in children, especially young children, is a challenge for anesthesiologists. The smallest available double-lumen tube (DLT) (26 Fr) has an external diameter (ED) of 8.7 mm and is equivalent to single-lumen tube (SLT) of 6 mm (ED: 8.2 mm) or 6.5 mm (ED: 8.9 mm). For this reason, DLT is not suitable for children <8 years of age. Additionally, performing an endobronchial intubation on a dependent lung can achieve an OLV for children <2 years of age. There is an anatomical variation in the take-off of the right upper lobe, which remains very close to the carina (≤1 cm) in children <8 years of age (15). Therefore, a right endobronchial intubation may not get efficient the right upper robe ventilation. A BB also can be performed to isolate an independent lung. In young children, especially children <2 years of age, an extraluminal placement of a BB is used commonly. In these cases, blocker compression and traction may damage vocal cords. Over distension of the cuff may injure bronchial walls. BB may not block the right upper robe completely for its higher opening location resulting in an incomplete collapse. Moreover, changes in body position and/or intraoperative traction may cause the displacement of SLT or BB, and result in the failure of OLV (15, 16). Compared with conventional mechanical ventilation, preserved spontaneous breathing can make lung collapse quickly after thoracic cavity opened and negative pressure disappeared. The likelihood of postoperative hypoxemia is greatly reduced due to avoiding residual muscle relaxants. Because of separation anxiety, preoperative sedation is essential in young children. Dexmedetomidine has been shown to produce stable sedation without respiratory depression and can provide analgesia with stable blood flow dynamics (17, 18). Children have large tongues and relatively narrow oropharyngeal cavities surrounded by soft structures. After sedation, the tongue is likely to retract and obstruct the airway. Using a laryngeal mask rather than a face mask can effectively open the airway and avoid airway obstruction caused by deep sedation. Furthermore, it can reduce sore throat, pharyngeal discomfort, hoarseness, laryngeal edema, vocal cord injury, and recurrent laryngeal nerve paralysis as Janík et al. describe (19). Ultrasound-guided PVNB technique is well-accepted and has associated advantages, including high safety, high success rate, and less related complications. In recent years, many studies have shown that the analgesic effects of PVNB and thoracic epidural block are equivalent (20, 21). The former only blocks the sympathetic nerve on the surgical side; has little effect on the normal physiological functions; does not inhibit the cardiac sympathetic nerve, blood vessels and myocardium contractile function; and is less likely to affect hemodynamics; consequently, it has achieved better results in anesthesia for thoracic surgery (22). This study showed that general anesthesia with PVNB combined with a laryngeal mask could be performed in pediatric thoracic surgery. This technique could produce complete analgesia with fewer stress reactions, stable vital signs, reduced perioperative opioid dosages, and no residual muscle relaxation. Gastrointestinal reactions after the operation were minimal. It was conducive to intestinal peristalsis and accelerates the recovery of digestive system, thus allowing patients to start eating earlier. Additionally, this method reduced the length of hospital stay after surgery and the total hospitalization expenses. Spontaneous breathing combined with PVNB was safe and feasible in infants and young children for VATS. This result was consistent with Wei's research in 3–8 year old children (12).

The bronchial blocker used for OLV is often displaced due to body position change and intraoperative traction, which affects the lung collapse on the operation side. In the intubation group in this study, displacement of tracheal blocker caused the failure of OLV in six patients, since the adjusting the blocker position was difficult. Thus, TLV had to be used for the operation. In the non-intubation group, the success rate of OLV established through iatrogenic pneumothorax under spontaneous breathing was high. During OLV, in addition to preventing hypoxemia and ventilator-related lung injury, attention must be paid to preventing CO_2_ retention (23). Compared with the intubation group, the non-intubation group had a higher intraoperative minimum SpO_2_ and a lower incidence of hypoxia. In the lateral decubitus position, patients in the non-intubation group can preserve spontaneous breathing and diaphragmatic movement. Thus, the incidence of atelectasis is low, and there is no significant imbalance in the ratio of ventilation to blood flow (5, 15). To avoid mediastinal flutter that may result in heart and respiration disfunction, we applied an iatrogenic pneumothorax with a low pressure of 6–12 mmHg. In addition, the absence of stimulation from the tracheal tube reduces airway stress and secretion production. There was no significant difference in the maximum P_ET_CO_2_ during surgery between the two groups, and there was no accumulation of CO_2_. This is mainly because the operation time was short, and breathing could return to normal soon after the operation (24)). However, due to prolonged one-lung spontaneous breathing or mediastinal flutter during the operation in the non-intubation group, six children (24%) had a short-term failure to effectively maintain blood oxygen saturation. For patients with spontaneous breathing, mediastinal flutter may occur due to the pressure differences between the left and right sides of the chest cavity after the chest is opened (24). In this study, one patient (4%) had severe coughing and mediastinal flutter. The operation continued after anesthesia was deepened, and the patient was switched to anesthetic machine-assisted ventilation. Respiratory management is most critical in this technique. The maintenance of oxygenation depends not only on whether the airway is open but also on the compression of the contralateral lung, the underlying lung disease, and airway secretions when patients are in the lateral decubitus position. Since the thoracic rib support ability of infants is low, the healthy lung is compressed in the lateral decubitus position, and its function can be greatly impacted. Due to the small diameter of the airway, a small volume of secretions or blood can cause a sharp increase in airway pressure, which can dramatically increase the breathing workload and affect anesthesia management. Therefore, it is necessary to strictly control indications and perform a comprehensive evaluation before surgery. The use of non-intubated anesthesia should be determined based on the technical capabilities of the anesthesia and surgical teams, monitoring and treatment conditions, surgical methods, disease types, individual differences in patients, and the actual conditions of various medical institutions (19). It is not applicable for patients who are undergoing complicated surgery that may involve a prolonged operation, massive blood loss (19), and the occurrence of mediastinal flutter. Appropriate sedation and analgesia during the operation should be performed with strict monitoring and enhanced management, and endotracheal intubation should be always prepared and ready for use if necessary.

Several limitations should be considered. First, this is a retrospective study so patients’ characteristics may result in severe selection bias. There are many respiratory parameters of spontaneous respiration, and mechanical ventilation cannot be collected completely. For a retrospective study, we collected all information from medical records. Regretfully, most of the time, the medical records cannot offer detailed information of the operation. It only mentions which side or lobe of lung was resected but not specific segments. Second, the duration of lung segmentectomy is short so we cannot draw a conclusion that non-intubated general anesthesia with preserved spontaneous breathing combined with PVNB is feasible for other thoracic surgeries. Third, there is not a reliable method to monitor the depth of anesthesia for young children. The BIS monitor is recommended for children over 2 years old commonly (25). Hence, the value of BIS to be monitored needs more studies. Furthermore, only patients classified as ASA I or II were included in the present study, and there are insufficient data about patients with perioperative lung dysfunction. According to Janík et al., not all patients are suitable for such a procedure, especially those with severe emphysema, obese patients, and those with a problematic oropharyngeal configuration—Mallampati score (19). The indication for non-intubated anesthesia techniques needs more precise study, especially about perioperative lung function (26).

## Conclusion

Non-intubated general anesthesia with preserved spontaneous breathing combined with PVNB is a safe and feasible lung separation technique in infants and young children for VATS. It can provide good exposure during thoracoscopic surgery, can avoid the complications associated with mechanical ventilation, and is prone to quick postoperative recovery and fewer postoperative complications. It can promote rapid rehabilitation and shorten the length of hospital stay, thus reducing medical costs. However, strict control of indications and anesthesia management by experienced anesthesiologists are the keys to success. For some limits in this study, prospective studies should be encouraged to verify the results by compatible surgery methods and refined airway management.

## Data Availability

The raw data supporting the conclusions of this article will be made available by the authors, without undue reservation.
